# Vanished Kidney by Pheripheral Nerve Seath Tumor: A Rare Case Report

**DOI:** 10.5812/numonthly.8029

**Published:** 2013-05-25

**Authors:** Anand Rai Bansal, Mahavir Singh Griwan, Yayathi Rajan Karthikeyan, Santosh Kumar Singh

**Affiliations:** 1 Department of Surgery and Urology, Sharma Postgraduate Institute of Medical Sciences, Rohtak, India

**Keywords:** Nerve Sheath Neoplasms, Retroperitoneal Space, Kidney

## Abstract

Malignant peripheral nerve-sheath tumor (MPNST) is a high-grade malignant tumor of ecto-mesenchymal origin comprising approximately 10% of soft-tissue sarcomas. They tend to occur associated with neurofibromatosis or sporadically. Here we report a MPNST at an extremely rare location and behavior causing disappearance of the entire kidney. Patient evaluation included clinical, biochemical and radiological studies before treatment. Histological study revealed the rare diagnosis. Patient was asymptomatic at six-month follow-up after treatment.

## 1. Introduction

Malignant peripheral nerve sheath tumor (MPNST) is derived from Schwann cells of major or minor peripheral nerve branches or from the sheath of peripheral nerve fibers (1). Most of these tumors arise on the trunk, extremities, or the head and neck region. MPNSTs arising from the abdominal cavity are extremely rare (2). The case alone with its management is hence being reported in view of extremely rare location and a rare behavior causing disappearance of the entire kidney in order to contribute to medical knowledge.

## 2. Case Presentation

**Figure 1.**
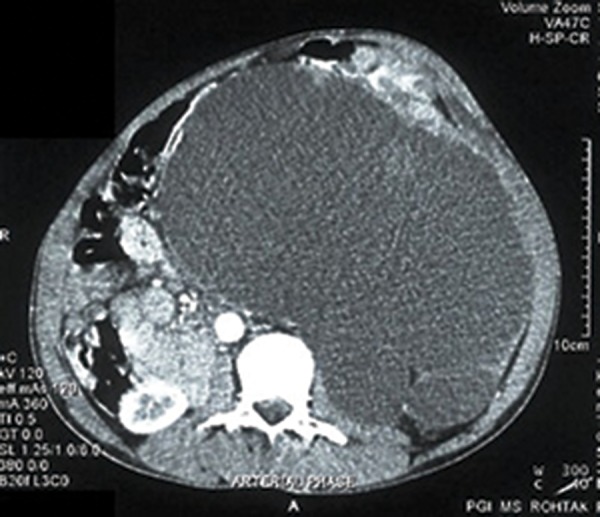
CECT Showing the Tumor and Right Kidney but Left Kidney Could Not Be Visualised

**Figure 2.**
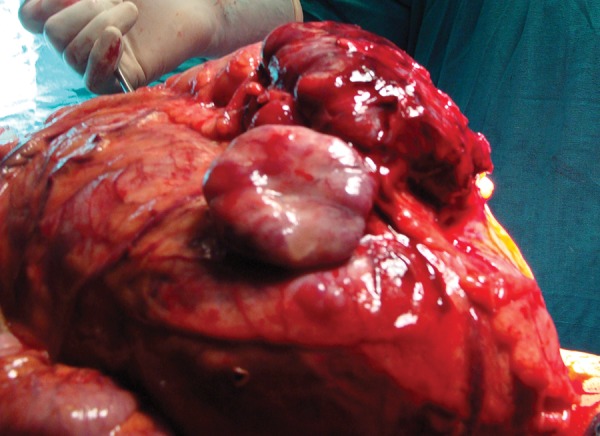
Intra Operative Tumor

A 55-year-old male reported with complaints of a gradually enlarging lump in the left side of the abdomen over a period of 5 months associated with dull aching pain and occasional vomiting. On examination there was a huge mass of around 25 × 20 cm spanning the whole abdomen with an irregular surface and variable soft to firm consistency. The ultrasonography of the abdomen suggested a large multiloculated cystic mass occupying the whole abdomen and pelvis, right kidney was normal but the left kidney could not be visualized. Contrast enhanced computer tomography of the abdomen showed a homogenous non enhancing retroperitoneal mass measuring approximately 17 × 21 cm with a mixed echogenic pattern in its anterolateral region (Figure 1). The cystic mass occupied the whole of the abdomen displacing the pancreas and left ureter anteriorly and across the midline. A focus of calcification was seen at the pheriphery of the lesion. Left kidney and its pelvicalyceal system was not visualized. There was a loss of fat plans with the adjacent gut loops. An exploratory laparotomy was done and a large cystic mass with solid areas weighing around 7 kg was found in the left side of the abdomen adherent to the distal transverse colon, splenic flexure and the descending colon (Figure 2). There was no identifiable left kidney. The tumor was resected en mass along with the adherent large bowel and a primary colo-colic anastamosis was done. The patient had an uneventful post operative course and was followed up for six months without recurrence.

**Figure 3.**
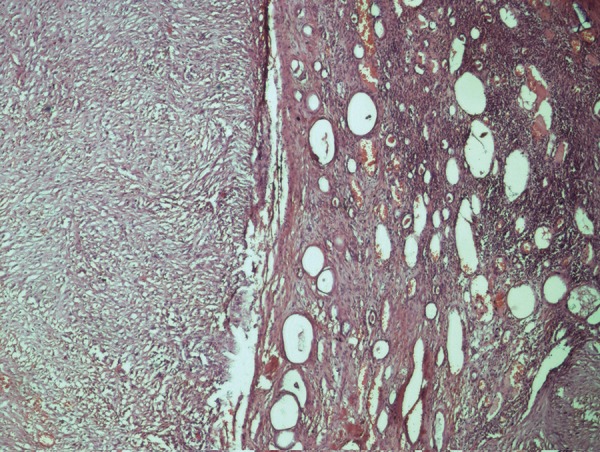
NSE Positivity

**Figure 4.**
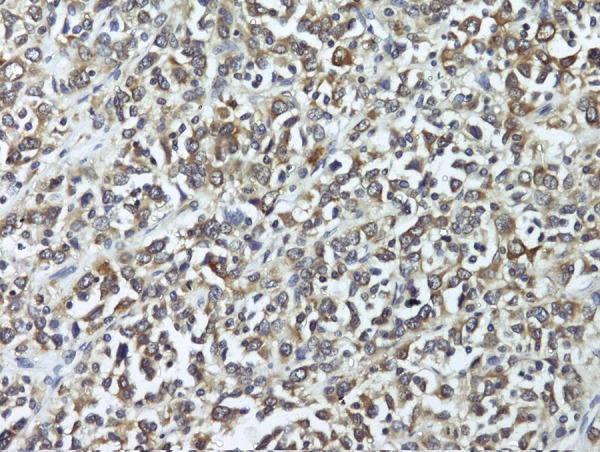
Kidney With Adjoining Tumor

The specimen grossly revealed 29 × 20 × 15 cm mass with marked congestion and a multinodular external surface, while the cut section revealed a multicystic mass with solid areas. Segment of large intestine measuring 5 cm was attached to it with no kidney identified grossly. Microsections showed a biphasic (epithelial and mesenchymal differentiation) malignant tumor with solid and cystic components infiltrating through the wall of the adherent bowel. However, the resection margins were free from the tumor. One of the sections showed a small amount of residual renal tissue at the periphery indicating that by and large the kidney was replaced by the tumor (Figure 3). The immunohistochemistry panel revealed CD34, CD117, calretinin, desmin, LCA, synaptophysin and chromogranin negativity and, NSE positive (Figure 4), patchy bcl2 positivity in ganglion cells and patchy EMA positive membrane. Hence the diagnosis of a retroperitoneal MPNST was made based on the clinic pathological co correlation.

## 3. Discussion

MPNST is a rare variety of soft-tissue sarcoma of ectomesenchymal origin accounting for up to 5-10% of all soft-tissue tumors and up to 50% of this is associated with Neurofibromatosis type 1 (NF 1). The estimated incidence of MPNST in NF 1 is 2-5 % as compared to 0.0001% in general population (3). Among the patients with NF1 MPNST is the leading cause of mortality. World Health organization (WHO) coined the term MPNST replacing previous heterogeneous and often confusing terminology such as malignant schwannoma, malignant neurilemmoma and neurofibrosarcoma for tumors of neurogenic origin and similar biological behavior. These tumors often create diagnostic problems because of their cellular origin and histopathological similarities with other spindle cell sarcomas like monophasic synovial sarcoma, leiomyosarcoma and fibrosarcoma. A combination of gross and microscopic findings along with immunohistochemical studies is commonly used to diagnose a case of MPNST. The pathologic diagnosis of MPNST is facilitated by features such as palisading arrangement, nuclear atypia, bizarre giant cells and staining analysis of such tumors reveals spindle cells with a fascicular pattern (2). In most instances, the tumors display fascicles of spindle cells woven into a herringbone pattern with varying degrees of mitosis and necrosis. However, it is not always possible to demonstrate the origin from a nerve, especially when it arises from a small peripheral branch. This point was exemplified in a series by Nambisan et al., in which nerves could not be identified in 61% of cases of MPNST. Still, there are several other distinct features, such as proliferation of tumor in the sub-endothelial zones of vessels with nepotistic cells herniation into vessel lumen and proliferation of small vessels in the walls of the large vessels, which are very characteristic features of MPNST (4). Histological and immunohistochemical markers specific for MPNSTs are not available. The S100 protein is the antigen most commonly used to identify nerve sheath tumors of various types (2). Another interesting clinical feature of this tumor is multifocality and development of second primary tumors of same histology (4).

These tumors arise from major or minor peripheral nerve branches or from the sheath of peripheral nerve fibers. Most of these tumors arise on the trunk, extremities, or the head and neck region. MPNSTs arising from the abdominal cavity are very rare. The retroperitoneum as a location is extremely rare and till date only three cases have been reported in literature (5). Radical surgical resection is the treatment of choice in MPNST. A good three-dimensional clearance is mandatory for a successful outcome. Routine nodal dissection is not indicated. However, when a major nerve is identified, the cut end should be sent for frozen section to assess the tumor free margin of the resection. MPNSTs are generally considered chemotherapy and radiotherapy resistant tumors. In view of the rarity of this entity and conflicting reports, it is difficult to define the role of radiation in the management of MPNSTs. Currently, postoperative radiotherapy is recommended by oncology consensus group as part of a uniform treatment policy for MPNSTs, much like other high grades soft-tissue sarcomas despite having clear surgical margins. MPNST has the highest recurrence rate of any sarcomas and adequate initial treatment gives the best chance of survival. Despite aggressive combined radiation and systemic chemotherapy, the 5-year survival rates for MPNSTs range from 35% to 50%. The current recommendation is that this therapy be reserved for recurrent tumors, suspected residual microscopic disease, and high-grade tumors. In this case the kidney was not identifiable as it had been completely replaced by the tumor, causing the kidney to vanish from the abdomen. A large retroperitoneal mass with an unidentifiable kidney or a retropenitoneal mass in a patient with neurofibromatosis, a malignant peripheral nerve sheath tumor should be considered as a possibility apart from primary renal neoplasms.
